# The Neanderthal niche space of Western Eurasia 145 ka to 30 ka ago

**DOI:** 10.1038/s41598-024-57490-4

**Published:** 2024-04-02

**Authors:** Peter M. Yaworsky, Emil S. Nielsen, Trine K. Nielsen

**Affiliations:** 1 Department of Archeology and Heritage Studies, School of Culture and Society, Aarhus University, Moesgård Allé 20, Building 4216, 8270 Højbjerg, Denmark; 2 Center for Ecological Dynamics in a Novel Biosphere, Department of Biology, Aarhus University, Ny Munkegade 114-116, 8000 Aarhus C, Denmark; 3 Moesgaard Museum, Moesgård Allé 15, 8270 Højbjerg, Denmark

**Keywords:** Ecological modelling, Ecological modelling, Climate-change adaptation

## Abstract

Neanderthals occupied Western Eurasia between 350 ka and 40 ka ago, during the climatically volatile Pleistocene. A key issue is to what extent Neanderthal populations expanded into areas of Western Eurasia and what conditions facilitated such range expansions. The range extent of Neanderthals is generally based on the distribution of Neanderthal material, but the land-altering nature of glacial periods has erased much of the already sparse material evidence of Neanderthals, particularly in the northern latitudes. To overcome this obstacle species distribution models can estimate past distributions of Neanderthals, however, most implementations are generally constrained spatially and temporally and may be artificially truncating the Neanderthal niche space. Using dated contexts from Neanderthal sites from across Western Eurasia, millennial-scale paleoclimate reconstructions, and a spatiotemporal species distribution model, we infer the fundamental climatic niche space of Neanderthals and estimate the extent of Neanderthal occupation. We find that (a.) despite the long timeframe, Neanderthals occupy a relatively narrow fundamental climatic niche space, (b.) the estimated projected potential Neanderthal niche space suggests a larger geographic range than the material record suggests, and (c.) that there was a general decline in the size of the projected potential Neanderthal niche from 145 ka ago onward, possibly contributing to their extinction.

## Introduction

Neanderthals (*Homo neanderthalensis*) are a close relative to anatomically modern humans^1–4^, that went extinct around 40 ka ago^5^. Neanderthals were originally thought to be a Western European phenomenon, but more recent research has shown that the Neanderthal range spanned across Eurasia reaching as far east as the Altai Mountains^6–8^. However, if the existing sample of the material record is spatially representative, Neanderthals were primarily a Western Eurasian species with a core area in Western Europe, but our understanding of the distribution of Neanderthals is biased by differential preservation in the material record. The material record is poorly preserved in areas where recent glaciations (i.e., Weichselian Glaciation) scoured away potential surface sites, and in areas where the geology is not conducive to preservation (i.e., lack of caves and poor conditions for stratified deposits)^9,10^. The absence of archaeological material resulting from the land altering dynamics of glaciations and poor preservation pose an interesting problem to archaeologists in understanding the past distribution of Neanderthals, and could lead to the conclusion that Neanderthals did not disperse more widely across Western Eurasia and into higher latitudes (> 55°N)^10–12^.

**Figure 1:**
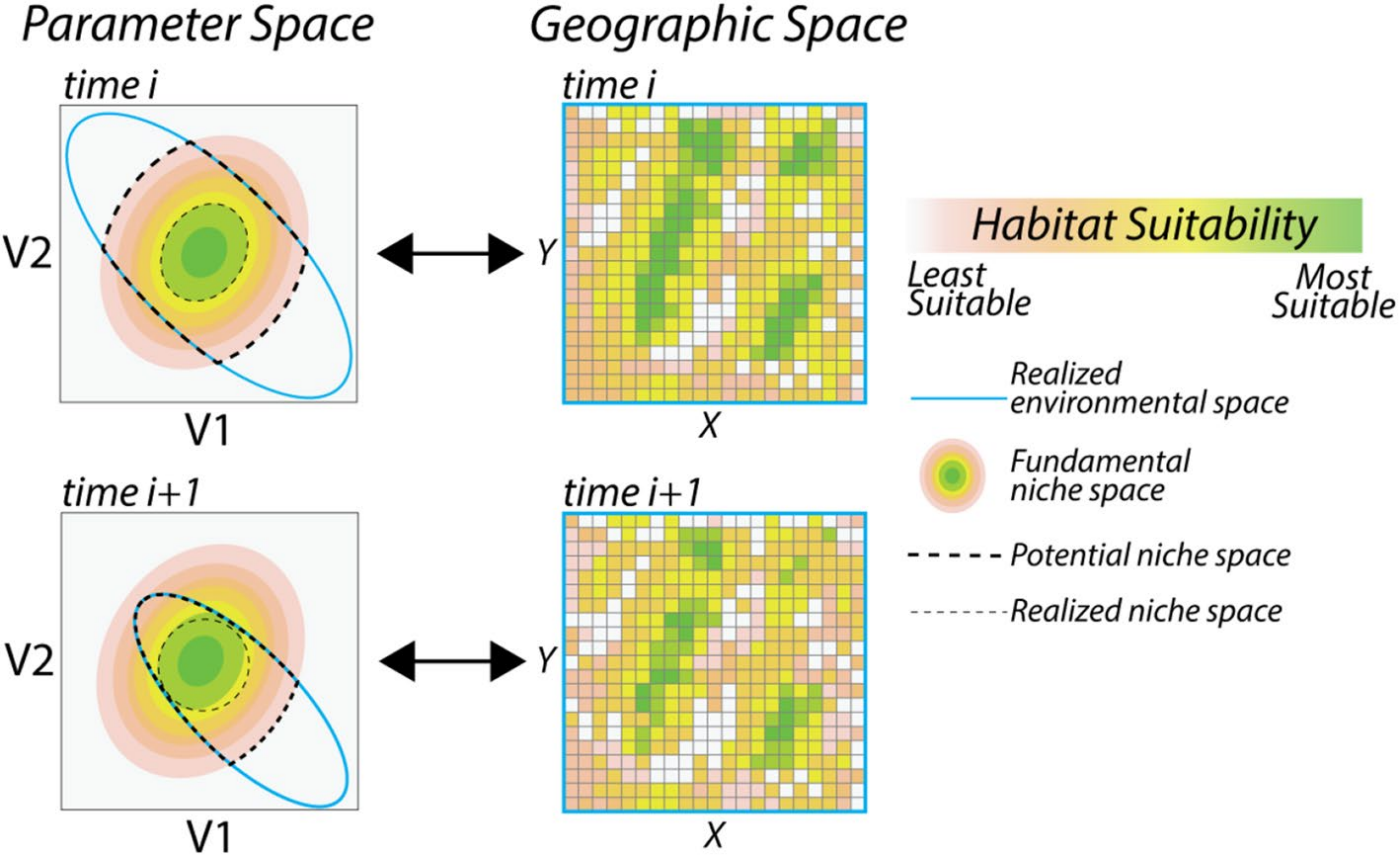
Illustrates the differences between key terms used in the paper, including the fundamental niche space, potential niche space, realized niche space, and realized environmental space. The left side of the figure illustrates the concepts in parameter space and the right side of the figure illustrates the concepts in projected geographic space using a simplified, two-variable example. Outlined in blue is the realized environmental space. The realized environmental space is composed of combinations of the two variables (V1 and V2). The fundamental niche space is the environmental space in which a species can survive and persist^18,20^. The fundamental nice space is represented by the topographic colors. Different combinations of V1 and V2 result in different suitability estimates, which we visualize with the color gradient representing habitat suitability. Note, that in parameter space the fundamental niche occurs outside the bounds of the realized environmental space, indicating that there are combinations of V1 and V2 that are suitable, but do not occur in the realized environmental space. The intersection of the fundamental niche space and the realized environmental space within parameter space defines the potential niche space (all colored cells in geographic space), which represents the subset of the realized environmental space in which a species can survive and reproduce^21^. The realized niche space is the portion of the potential niche space in which a species is observed (not shown in geographic space). In the figure, there is a shift in the realized environmental space between *time* *i* and *time* *i + 1*, indicating some change has altered the combinations of V1 and V2 present in the environment. The shift in the realized environmental space results in a shift in the potential niche space, which can impact the realized niche space, but the fundamental niche remains the same.

Past research has modeled the distribution of Neanderthals at both regional and continental extents with varying temporal scales and focuses^11,13–17^. Most commonly, researchers implement some form of correlative ecological niche modeling, which are approximations of a species ecological niche space built on relationships between environmental data and species’ observations^18^. There are several terms used to refer to correlative niche modeling, including eco-cultural niche models^19^, climate envelope models, habitat suitability models, and species distribution modeling^18^. Here, we use the latter, species distribution model. Central to species distribution modeling are concepts from ecological niche modeling theory, such as the *fundamental niche space*, *potential niche space*, *realized niche space*, and *realized environmental space* (Fig. 1). The fundamental niche space exists as an *n*-dimensional hypervolume defining an area in parameter space in which a species can persist^20^. The fundamental niche space of a species is often constrained by the realized environmental space, wherein at a specific point in time, the entire range of the fundamental niche space in parameter space may not be present in the realized environment. The intersection between the fundamental niche space and the realized environmental space is the potential niche space, which can be projected into geographic space^21^. The locations where a species is observed is the realized niche, which is a subset of the potential niche space often as a result of competition, displacement, predation, parasitism, symbiosis, dispersal constraints^18^, and density derived benefits and pressures^22^.

Adapting species distribution models to observations of past species is challenging, in that species distribution models were developed around species in the present observed in present environments, estimating the probability of a species’ occurrence or habitat suitability from the spatial variance in environmental conditions, while holding the environmental temporal variation constant. Applications of species distribution models with past observations of species in past environments necessitates that we incorporate the temporal variation present in the environments these species occupied, thus a spatiotemporal species distribution model^23–25^.

Specific to Neanderthal distribution research, to date there are no continental studies of the distribution of Neanderthals spanning from before the proliferation of Neanderthals in Europe (145 ka ago) to their extinction (~ 40 ka ago). There are two reasons for why such an ambitious model has not been implemented. First, there is a relatively small sample size of dated contexts at Neanderthal sites making it difficult to generate robust predictions. Second, the resolution of paleoclimate records, both spatially and temporally, with adequate time depth, did not exist until recently. Past implementations of species distribution models applied to Neanderthal biogeography have proven effective, but most compromise to overcome the two aforementioned issues by narrowing their spatial extent to more regional focuses and, either omitting or summarizing the temporal variation into lower resolution time steps (i.e., MIS stages, stadials and interstadials). The former has the potential to reveal important local trends, but the latter can severely cripple our understanding of temporal dynamics in the distribution of species, and both run the risk of artificially truncating estimates of the fundamental niche space^26,27^. The fundamental niche space of a species is best approximated from an ensemble of climate data across the temporal range of a species’ existence^24,27–29^. To adequately address questions pertaining to the distribution and adaptation of past species, we need to understand variation present at occupied habitats, both spatially and temporally, and incorporate that variation into a single model to better estimate the fundamental niche space.

**Figure 2:**
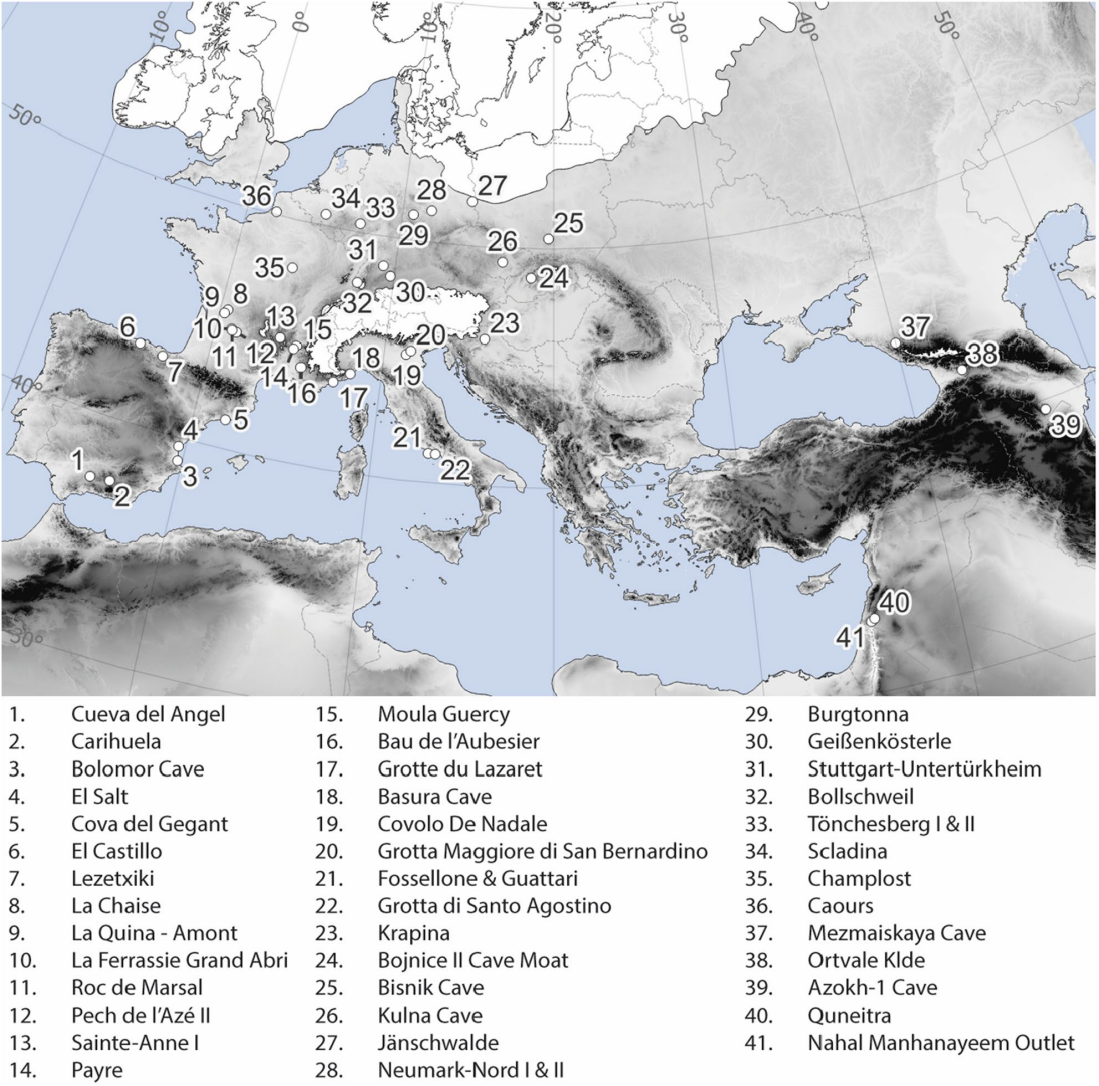
Shows the distribution of archaeological localities dating between 145 ka and 50 ka ago across Western Eurasia. The white areas indicate the maximum glacial extent during the Last Glacial Maximum (~ 20 ka ago). Neanderthal localities are distributed throughout Western Europe, with their northern extent abutting the southern extent of the ice sheets during the LGM. Note that several of the localities are grouped together due to their spatial proximity. Map data acquired from^99,100^ and created in QGIS v3.34^101^.

Here, using a spatiotemporal species distribution model, the *Role of Culture in Early Expansions of Humans Out of Africa* (ROCEEH: http://www.roceeh.net) *Database* (ROAD^30,31^) data for Neanderthal archaeological sites from Western Eurasia dating from 145 ka to 50 ka ago (Fig. 2), and spatially resolved paleoclimate records at a millennial-scale temporal resolution^32,33^, we estimate the fundamental climatic niche space^20,34,35^ and geographic distribution of the potential climatic niche space^18,21^ of Neanderthals for Western Eurasia from 145 ka to 30 ka ago.

Building on past research^23–25,36–41^, we construct the spatiotemporal species distribution model using Maximum Entropy^42–44^. Key differences in the spatiotemporal species distribution model from past model implementations are that:

1. The model uses predictor values from spatially and temporally resolved archaeological contexts, meaning that each archaeological observation has not only a location coordinate, but also a time coordinate derived from absolute dates.
2. The model uses spatially and temporally resolved background points, from which Maxent estimates the range of habitats available.
3. Neither time (i.e., year BP) nor space (i.e., latitude and longitude) are explicit predictors in the model. Rather, time and space are incorporated into the model through the spatial and temporal variation present in the predictor values derived from the archaeological and background observations.
4. We construct only a single model for predictions, which uses all archaeological observations and background observations, thus assuming stability in the fundamental niche space^25^ and that the same processes underlying behavior were present throughout time (i.e., foraging subsistence, uniform technological development, notwithstanding some variation^45^), leveraging the small sample size of available data. This is not to say that there were no changes in technology and material culture, but that these changes did not alter the fundamental niche of Neanderthals, thus allowing for their expansion into vastly different climate niches.
5. The predictions produced from the single model are spatially and temporally explicit allowing us to measure changes in the projected potential niche space over time and understand spatial and temporal trends at a finer resolution than ever before.

These key differences represent a significant improvement in species distribution models applied to past distributions of species over those that omit the temporal variation, and provide new, high-resolution insights to our understanding of the spatial and temporal distribution of Neanderthals throughout the Pleistocene and how the size and distribution of the projected potential climatic niche space changed in response to changing climates 145 ka to 30 ka ago.

By using a broader temporal and spatial extent, we hope to capture the range of realized environments encountered by Neanderthals between 145 ka ago and 50 ka ago to better understand the climatic spaces in which they lived. While specific processes have biased the spatial distribution of Neanderthal observations, we may still be able to derive a representative sample of the types of habitats Neanderthals occupied within parameter space, allowing us to accurately estimate the fundamental niche space. Doing so helps us overcome the spatial bias presented by the archaeological record, as an accurate estimate of the fundamental niche space can help us assess the suitability of a biased location, and better estimate, in the absence of material evidence, whether that location would have been suitable for Neanderthal occupation at some point in the past. Using estimates of the fundamental niche space and geographically projected potential niche space, we test (a) what regions of Western Eurasia were likely occupied during specific millennia, (b) when and why Neanderthals expanded into areas like Northern and Eastern Europe, and (c) whether there were significant changes in the size of the projected potential Neanderthal niche space which may have contributed to their eventual extinction.

We find that the projected potential Neanderthal niche space centered in Western Europe, the Iberian Peninsula, and the Adriatic coast aligning with previous studies^11^. During warmer interglacial and interstadial periods, the projected potential climatic niche space expanded, reaching a maximum 121 ka ago during MIS5e, expanding as far north as central Scandinavia and across Eastern Europe. Our temporal resolution allows us to estimate the evolution of the projected potential Neanderthal niche space during the post-Eemian climate deterioration and interstadial warmings of Brørup (MIS5c) and Odderadde (MIS5a), supporting notions of ephemeral Neanderthal dispersal into the higher latitudes where material evidence may be lacking^10^. We find that both Scandinavia and Eastern Europe appear to be areas with climates generally less suitable for Neanderthals, particularly during stadials. The results have important implications for understanding the general adaptive capacities of Neanderthals. Last, the general temporal trend in the projected potential Neanderthal niche size is decline. The projected potential Neanderthal niche in Western Eurasia peaks 121 ka ago during MIS5e and then gradually declines reaching an all-time low around 31 ka ago, roughly the time corresponding to estimates of Neanderthal extinction 42 ka to 36 ka ago^3,46–49^, suggesting changes in climate played an important role in Neanderthal extinction^5^.

## Results

Using the ROCEEH data for Europe and Asia dating between 145 ka and 20 ka BP (*n* = 7893), we pair each archaeological context (Supplemental 1) with its associated absolute dates (Supplemental 2–4) and remove duplicate observations and dates younger than 50 ka BP resulting in a dataset with 94 unique observations in Western Eurasia from 45 localities (Supplemental 5, see Fig. 2). Alongside the archaeological observations, we generate 100 random background points in each millennium totaling 9600 (see "Materials and Methods" Section; Supplemental 6).

**Figure 3:**
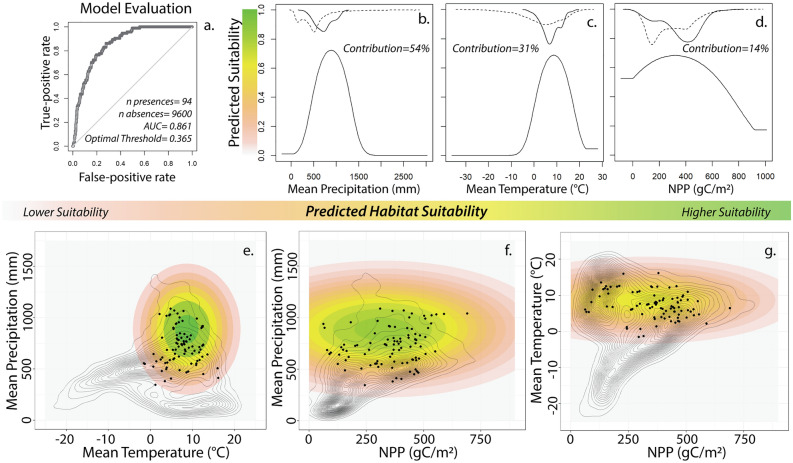
Shows the results of the final model. Panel a shows the receiver operating characteristic (ROC) curve illustrating the model’s ability for binary classification of presence/absence observations. Using the predictor variables and their relationship to habitat suitability for Neanderthals, we can characterize the fundamental Neanderthal climatic niche space in parameter space. Panels (**b**–**d**) show the response plots of our predictor variables (mean precipitation, mean temperature, and net primary productivity [NPP]) and each variables contribution to the model predictions. At the top of panels (**b**–**d**) are inverted density plots illustrating the distribution of values for background points (dash line) and presence points (solid line). Note that the solid line is shifted to the right in all cases. The y-axes of panels (**b**–**d**) are the predicted habitat suitability at the corresponding x-values while holding values for the other two variables at a constant. Panels (**e**–**g**) illustrate the fundamental niche space in parameter space by varying two variables at a time while holding the third variable at a constant. The colored contours broadly outline the fundamental niche in parameter space with a continuous estimate of suitability. The black dots indicate presence observations in parameter space, and the gray contours represent the density of background observations in parameter space, thus representing the realized environmental space. The outer limits of the gray contours broadly indicate the potential niche space in parameter space. Combined, panels (**b**–**g**) illustrate a bias in habitat selection for wetter, warmer, and higher NPP habitats.

Using archaeological presence and background observations, we construct the spatiotemporal species distribution model. Using the optimal model parameters for model selection (see Methods) the ten-fold cross-validation results for the selected model are consistent across the folds (*mean AUC* = 0.853; *sd AUC* = 0.041; *∆ AIC*_*c*_ = 0). We then construct the full model (*AUC* = 0.861; *TSS* = 0.581) (Fig. 3a). From the model, we estimate the fundamental climatic niche space of Neanderthals. Mean precipitation is the most important variable to the model (*contribution* = 54.6%), followed by mean temperature (*contribution* = 31.3%). Net primary productivity (NPP) is the least important variable *(contribution* = 14.1%). The fundamental Neanderthal niche space is characterized within parameter space as precipitation ranging from roughly 550–1250 mm peaking around 900 mm, a mean temperature centered around 8 °C and ranging from 2 to 15 °C, and NPP ranging from 0 to 900 gC/m^2^ (grams of carbon per square meter), peaking around 230 gC/m^2^ (Fig. 3b–d).

**Figure 4:**
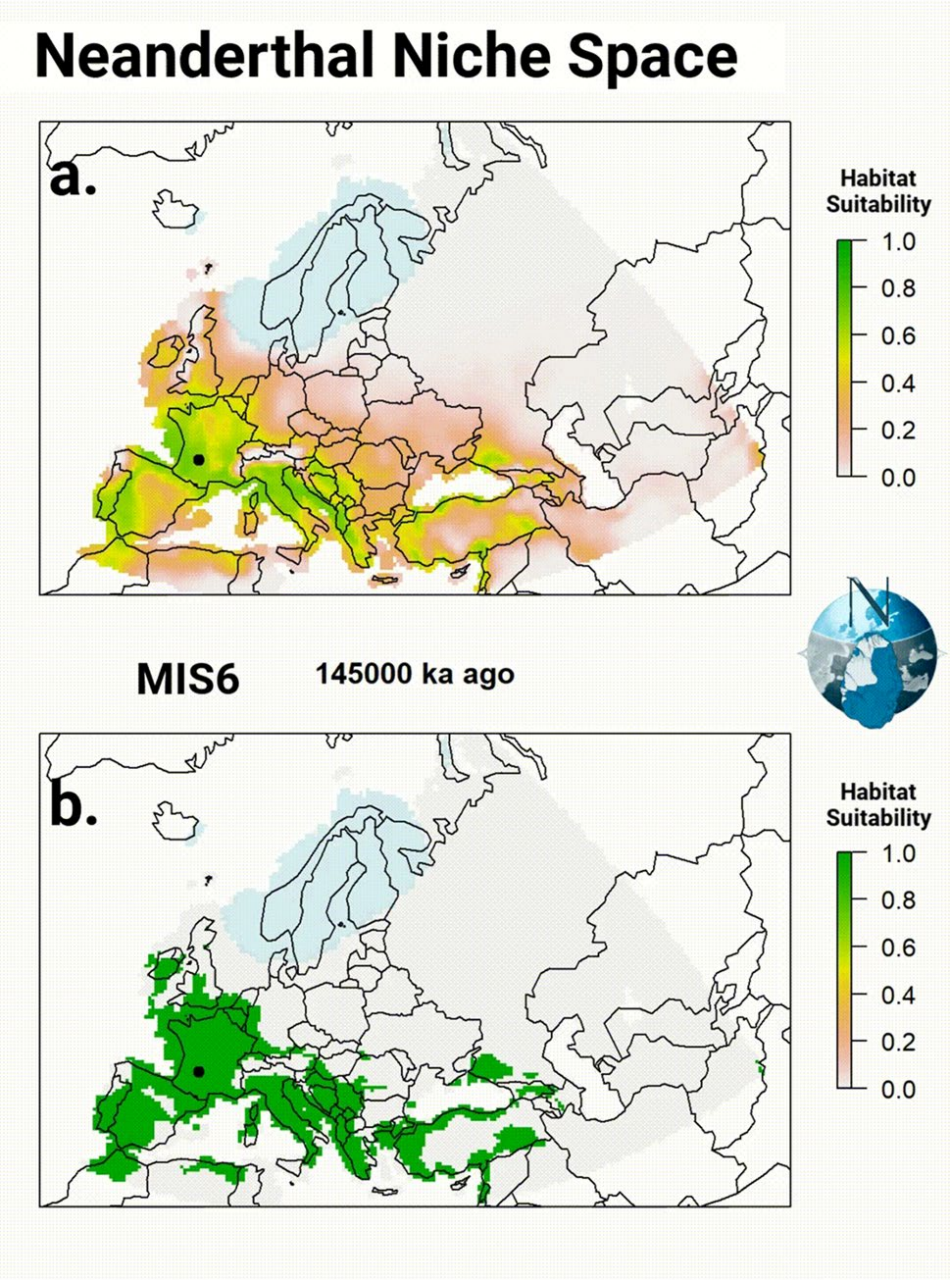
Figure 4 is composed of two animated GIFs showing the projected potential Neanderthal niche space (see Supplemental 7). The top panel shows the continuous predictions produced by the model when applied across the millennium-scale paleoclimate data^32^. The predictions range from 0 to 1 and can be interpreted as the climatic suitability for Neanderthals in that cell at each time step. The lower panel shows the binary predictions derived from the continuous predictions. We use the binary predictions as an estimate of the size of the projected potential Neanderthal climatic niche space. In both maps, archaeological observations occurring during the millennium are shown in black. Note that both sea level and glacial extent (shown in light blue) are incorporated into the predictions. The modern country map of Europe is overlayed to aid in interpretation^102^. The maps were created in R v4.3.3^84^ and the high-resolution animation can be found in Supplemental 7^85^.

**Figure 5:**
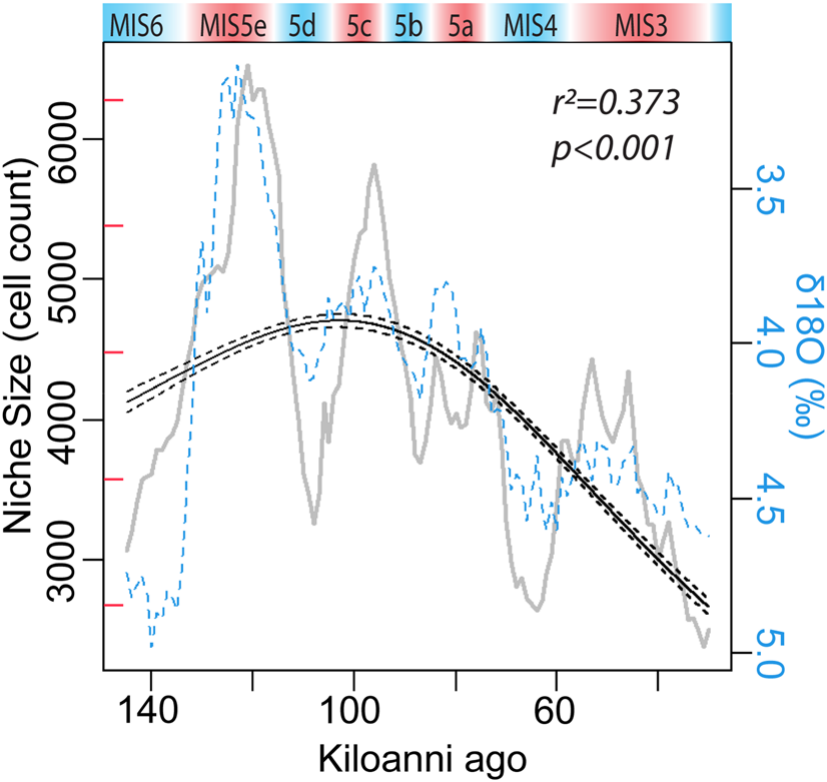
Illustrates the relationship between the size of the projected potential Neanderthal niche space relative to the year BP. The gray line indicates the millennial-scale change in the size of the projected potential Neanderthal niche. The blue dashed line represents changes in δ18O (Supplemental 9)^103^, with interglacial periods colored in red and glacial periods colored in blue at the top with their MIS labels. The black line is the GAMM model fit and 95% confidence intervals along with the model statistics. From MIS5e onwards, there is a general decline in the size of the projected potential Neanderthal niche space. The red ticks along the y-axis indicate one million square kilometer intervals from three million to seven million.

We then project our estimates of the fundamental niche space produced by the model over the millennial-scale paleoclimate data representing the realized environmental spaces to produce continuous estimates of the projected potential niche space and climatic suitability (Fig. 4; Supplemental 7). Using an optimal threshold value^50^, wherein the sum of the true-positive rate and true-negative rate are maximized (*Optimal Threshold* = 0.365; *Kohen’s kappa* = 0.0386), we convert the continuous estimates of climatic suitability to binary (presence/absence) to better refine our estimates of the projected potential niche space for Neanderthals (Fig. 4). Using the continuous and binary predictions, we can (a) estimate the mean climatic suitability for Neanderthals of Western Eurasia for each millennium, (b) estimate the size of the projected potential climatic niche space for each millennium, and (c) estimate the mean suitability of archaeological observations (Supplemental 8). Using a generalized additive mixed model (GAMM^51^), which accounts for the temporal structure within the data by specifying a correlation structure (corAR1), we test for change in the estimated size of the projected potential climatic niche space through time, from 145 ka to 30 ka ago and find a significant trend (*r*^2^ = 0.373, *edf* = 2, *p* < 0.001), in which as time progresses, there is a general pattern of contraction from the Eemian onwards (Fig. 5). All analyses and results can be found in Supplemental 9.

## Discussion

The distribution of Neanderthals has generally been understood through direct fossil, cultural, and genetic observations using static map representations of their maximum range (e.g.^6^). A known problem with this approach, is that the archaeological record of Neanderthals suffers from taphonomic bias, which has potentially accentuated patterns of ‘core’ and ‘periphery’ in Neanderthal occupations and quite possibly even erased the majority of evidence in the most northern latitudes of Eurasia^10,12^. As such, there are a limited number of ways in which we can build an understanding of the true geographical range of Neanderthals, and how this range would have changed as a result of the climatic fluctuations during the Pleistocene.

Building on the environmental variation present in Neanderthal observations attributable to specific locations in space and time, we attempt to overcome issues of small sample size and spatial bias in taphonomic processes by estimating the fundamental niche space. Using the spatiotemporal species distribution model, we infer the fundamental niche space of Neanderthals and estimate the size and distribution of the projected potential niche space of Neanderthals from before their proliferation ~ 145 ka ago, to after their extinction ~ 30 ka ago. In doing so, we are able to estimate at a millennial temporal scale the potential spatial extent of the Neanderthal range. While a specific location in space may suffer from severe taphonomic biases, the environmental conditions present at that location during a given time are potentially represented elsewhere in time in a spatial location where the taphonomic biases are less severe. While a species is not observed in a specific spatial location, it may (or may not be) observed in a specific environmental setting. So, if the range of environmental settings (combinations of the predictors) have been adequately surveyed, then we can estimate and project the fundamental niche space into areas lacking observations due to taphonomic biases. The underlying assumptions are that we have adequately inventoried the potential parameter space^36^ and that there is temporal stability in the fundamental niche space of Neanderthals. The former is difficult to evaluate as the underlying inventory data are unavailable, and the latter is supported by our results (see "Materials and Methods" Section). The process of using the fundamental niche space in overcoming potential taphonomic biases is best illustrated in our geographic projections of the potential niche space (Fig. 4), wherein during certain periods we observe the projected potential niche space for Neanderthals at latitudes farther north than material evidence exists due to known taphonomic biases^10,12^.A key finding is that the projected potential Neanderthal niche space extends farther into the northern latitudes than the material record would suggest^52^. While species distribution models can only estimate the fundamental and projected potential niche spaces of Neanderthals, the question remains as to the realized niche space of Neanderthals. The threshold-based estimate of the Neanderthal niche space (see Fig. 4) is our best estimation for the extent of the Neanderthal range, given the existing data, however there may have existed water barriers inhibiting Neanderthal populations from expanding into some areas. Building evidence indicates a connection between the North Sea and the Baltic Sea which separated Jutland and Fennoscandia from Europe during parts of the Eemian^53^. The depth and duration of the water feature separating Jutland from continental Europe, which is central to determining whether it acted as a persistent barrier to Neanderthal dispersals into Scandinavia during the Eemian, are unknown. However, the water feature is generally believed to be shallow and short lived (127 ka to 120 ka ago)^54^ limiting its potential effect as a barrier to Neanderthal expansion. With no prolonged major barriers preventing the expansion of Neanderthal populations into the northern and eastern projected potential niche spaces, the absence of material finds is potentially a result of the glacial ice sheets and poor conditions for preservation.

The model estimates of the fundamental niche space generalize the climate envelope Neanderthals lived in (Fig. 3), and how changes in climate during the Pleistocene impacted the realized environmental space and the projected potential Neanderthal niche space. The characteristics of the fundamental Neanderthal niche space (Fig. 3) do not suggest a specifically cold-adapted species^55,56^, but instead a species constrained by the climatic conditions of the Pleistocene. The results indicate a biased habitat selection, in which Neanderthals preferred habitats that were both warmer, wetter, and more productive than the average habitat available in Western Eurasia. Precipitation and temperature appear to be the most important constraints on habitat selection and preliminary analyses suggest an overlap in the fundamental niche space with later anatomically modern humans^5,29,57–59^. A comparative analysis of the two niche spaces, using observations on prehistoric hunter gatherers is needed, as shifts in subsistence to farming may bias the human niche space towards the warmer niche spaces of domesticates^60^.

The limiting effects of climate on foraging population in Europe after the Last Glacial Maximum are well observed^61–63^. As such, the changes in the size of the projected potential Neanderthal climatic niche space likely correlate with changes in overall population size and distribution, wherein as climatic conditions improve, population size increases. Figure 4 illustrates the general spatial and temporal trends in the projected potential Neanderthal climatic niche space from 145 ka to 30 ka ago. At the beginning of the sequence 145 ka ago, the projected potential Neanderthal niche space is concentrated in Western Europe, and along the Italian Peninsula and along the Adriatic Sea with pockets extending east along the Mediterranean into the Aegean and Black Sea. After 145 ka ago, the projected potential niche space rapidly expands as climate conditions warm 130 ka ago during the transition from MIS6 to MIS5. The size of the Neanderthal projected potential niche space peaks at 7.3 million km^2^ around 121 ka ago, corresponding to changes around the peak of the Eemian interglacial period MIS5e^64^. This warming leads to suitable conditions for Neanderthal expansion outside of Western Europe in the northern latitudes of central Fennoscandia and into Eastern Europe. Then, as MIS5e transitions to the cold period of MIS5d (Herning stadial), the projected potential niche space drastically contracts confining the extent of the projected potential niche space to areas of Europe similar to that at the end of MIS6. The expansion of the projected potential niche space and associated range in the warm Eemian Interglacial is supported by increasing archaeological site density and high genetic diversity^65–68^. Less known is the effect of the immediate post-Eemian climate oscillations on the Neanderthal population and range. Between 116 ka and 71 ka ago (MIS5d-a) there were two major periods of expansion and contraction. After the initial cooling of the Herning stadial (MIS5d), the Brørup interstadial (MIS5c) again permits the projected potential niche space to expand north into southern Scandinavia and large parts of Doggerland. This cyclic pattern is mirrored (with contraction followed by expansion) in the Rederstall stadial (MIS5b) and Odderade interstadial (MIS5a), but with an overall smaller expansion falling in the later part of the Odderade warming. The recurring suitability for Neanderthal occupation in the lowlands of the North European Plain sheds important light on this often overlooked early Weichselian period between MIS5e and MIS3, and aligns well with recent findings at sites like Lichtenberg^15^.

The transition from MIS5a (the Odderade interstadial) to MIS4 (~ 75 ka ago), is roughly the last time that the projected potential climate niche space expanded north of southern Scandinavia and into northeastern Eurasia. After 65 ka ago, Neanderthal expansion is minimal and their projected potential climatic niche space is confined to Western Europe, parts of the Iberian Peninsula, and along the Italian Peninsula and Adriatic, with pockets along the Eastern Mediterranean. Our model suggests that Eastern Europe is relatively unsuitable for Neanderthals during stadial events throughout the Upper Pleistocene, indicating climate differences between Western Europe and continental Europe persisting throughout the Pleistocene. While there may have been greater abundances of megafauna in Eastern Europe within the Mammoth Steppe^69^, this does not appear to have been a draw for Neanderthal populations, possibly due to the abundances of higher ranked prey species, like reindeer, elsewhere^28,63^. Our model supports that refugia for Neanderthals during glacial periods existed, primarily in Western Europe and Iberia^70^, and along the Italian Peninsula, the Adriatic & Mediterranean Coasts^71,72^, but there is no indication in our model for the suggested northern or northeastern refugia^69,73,74^.

After MIS5e, there is a gradual decline in the projected potential niche space size and the mean climatic suitability of habitats for Neanderthals (Fig. 5). With each successive stadial-interstadial cycle, the projected potential climatic niche space decreases in size, reaching its lowest point at 31 ka ago (2.7 million km^2^) during the onset of MIS2. The projected potential niche space size around the suspected extinction of Neanderthals 36 ka to 42 ka ago^46–48^ is most similar to that of the projected potential niche space size 65 ka ago between MIS4 and MIS3, MIS6 ~ 153 ka ago, and MIS8 ~ 270 ka ago (see Supplemental 9). The difference between MIS3 and MIS2 is most likely that the projected potential climatic niche space during MIS3 never expanded to the broad extents of previous periods, suggesting that Neanderthal populations may have been on a general decline due to habitat loss as far back as MIS5e. Mitochondrial DNA points to a significant Neanderthal population bottleneck occurring prior to the arrival of anatomically modern humans^75^. The results suggest that climate potentially played a significant role in the extinction of Neanderthals in Western Eurasia, simply due to their projected potential climatic niche space shrinking, however, the Neanderthal niche reached similar low points during MIS6 and MIS8 and Neanderthals did not go extinct then. This may point to a confounding factor of *Homo sapiens* migrating into Western Eurasia during MIS2. Dispersing *Homo sapiens* populations likely impacted Neanderthal populations^76^. The latest period of expansion for the projected potential Neanderthal climatic niche space occurred during MIS3, peaking ~ 53 ka ago at 5 million km^2^, roughly around the same time anatomically modern humans begin dispersing into Western Eurasia^73,77–80^. The decline and eventual extinction of Neanderthals is likely attributable to both changes in climate^5^ and complex interactions with migrating modern humans^5,81–83^, but, climate was shifting and the size of the projected potential Neanderthal niche space, and perhaps populations as well, peaked in MIS5e 121 ka ago, and had been declining ever since.

## Materials and methods

All analyses are performed in R^84^. The data and methods are available as supplementary material on Zenodo^85^.

### Data

From the *Role of Culture in Early Expansions of Humans Out of Africa* (ROCEEH: http://www.roceeh.net) *Database* (ROAD^30,31^), we requested all archaeological observations estimated to date before 20 ka BP in Europe and Asia. The query resulted in 7893 observations of archaeological contexts (Supplemental 1). The 7893 archaeological observations were missing associated dates derived from absolute dating methods. We requested the absolute date data tables from ROCEEH for our archaeological observations. These data come in three tables corresponding to column names in the archaeological data (Supplemental 2–4).

In order to estimate the date for each archaeological observation, we begin by pairing each archaeological observation in Supplemental 1 with the absolute dates in the three dates tables (Supplemental 2–4). Absolute dating methods present in these data include radiocarbon dates, thermoluminescence, electron spin resonance, optically stimulated luminescence, and uranium series. Most archaeological observations have several dates associated with them, sometimes derived by different methods. In order to incorporate all the dates data for an observation into an estimate of occupation year, we begin by calibrating the radiocarbon dates of an observation, where present, using the Intcal20 calibration curve^86^ and the package *rcarbon*^87^ to create probability density functions. Next, using the mean and error provided in the dates data for the non-radiocarbon absolute dating methods, we derive probability density functions. We sum the probability distribution functions creating a single probability distribution. As some dating methods have smaller error estimates than others, the method essentially weights dates associated with narrower error ranges, like radiocarbon dates, higher. Then, we identify the year BP that corresponds to the peak of the summed probability distribution function, which represents the most likely date with the data provided. Last, we measure the area of the probability distribution function under the curve within 5000 years of the estimated date to estimate the confidence in the assigned year (see Supplemental 5). The estimated date and confidence estimate are recorded for each archaeological observation. Archaeological observations without any absolute dates are not assigned a date estimate. Then, using the estimated date, we round each archaeological observation to the nearest millennium. Next, we remove observations younger than 50 ka BP (to avoid any non-Neanderthal *Homo* observations) and older than 145 ka BP, and observations east of 70° E and south of 35° N, bounding our observations spatially to Western Eurasia. Finally, we remove observations that are duplicated in both time and space. These are archaeological observations located at the same site as defined by spatial location (latitude/longitude) and assigned to the same millennium. After removing these data, we are left with 94 archaeological observations at 45 localities (Supplemental 5; Fig. 2).

### Background points

Background points are used to characterize the environments in a study region^88^ by understanding the distribution of values in the environment in parameter space. Because our model is a spatiotemporal model, our selection of background points needs to incorporate the spatial and temporal variation of our predictor variables. To derive an accurate estimation of the environmental space and incorporate the temporal and spatial variation, we randomly generate 100 background points in each millennium in R while avoiding cells under water, cells under ice sheets, and cells outside the project extent (Supplemental 9). The process results in the generation of 9600 background points across all time intervals (Supplemental 6).

### Variables

We use paleoclimate variables derived from a global terrestrial climate dataset based on HaDCM3 climate simulations of the last 120 ka^89^, that were then downscaled and extended back to 800 ka ago using linear regression^32^, and accessed through the R packages *pastclim* and *pastclimdata*^33^. We select variables from the bioclim data^90^ as well as net primary productivity, and biome. These data are available on a millennium time scale at 0.5-degree spatial resolution. The variables include mean temperature (bio01), min temperature (bio05), max temperature (bio06), mean temperature during warm quarter (bio10), mean temperature during cold quarter (bio11), mean precipitation (bio12), mean precipitation during cold quarter (bio19), net primary productivity (npp), and biome. These data are masked so as to account for both changes in sea level and glacial extent for each millennium^32^. The variables are a climatic subset of a wider array of environmental variables that are believed to constrain the Neanderthal niche space, or the conditions within which Neanderthals can persist.

### Extraction

To extract values associated with each archaeological observation and background point, we orient them not only in their correct spatial location, but also their correct temporal location. Doing so allows us to incorporate the spatial and temporal variation in the environment throughout time. After properly orienting the archaeological observations and background points in time and space, we extract their underlying cell values from the predictor variable rasters (see Supplemental 9). All spatial data were transformed to the Lambert azimuth equal-area projection (EPSG:3035).

### Variable selection

After extracting the values for archaeological observations and background points, we use a correlation matrix to select a subset of variables for our spatiotemporal species distribution model. We select a subset of variables based on their correlation coefficient, with preference for variables with a weak correlation (*Pearson’s r* > 0.7; see Supplemental 9)^23,36,91^. The process results in the selection of three variables, mean temperature (bio01), mean precipitation (bio12), and net primary productivity (NPP). These variables likely acted as limiting factors on Neanderthals land use patterns in similar ways as they did for *H. Sapiens* during the Pleistocene in Western Eurasia^61^. Biome was originally included but did not contribute to model predictions and was therefore excluded in future iterations. Variable importance was determined using *maxent.jar* to measure *% contribution* of each variable.

### Niche stability

To test for temporal stability in the fundamental climatic niche^25^ for Neanderthals, we constructed six generalized additive models^92^, three modeling the distribution of variable values at background points through time, and three modeling the distribution of variable values at observation points. For mean annual temperature, we find no significant change in values across archaeological observations (*k* = 3, *edf* = 1, *p* = 0.73) even though there are changes in values observed at background points (*k* = 3, *edf* = 2, *p* < 0.001). For mean annual precipitation, we find no significant change in values across archaeological observations (*k* = 3, *edf* = 1, *p* = 0.83) even though there are changes in values observed at background points (*k* = 3, *edf* = 1.9, *p* < 0.001). For mean annual NPP, we find a significant change in values across archaeological observations (*k* = 3, *edf* = 1.7, *p* < 0.001) beginning about 70 ka ago (also observed in^37^), wherein Neanderthals are occupying habitats with lower NPP values than before. The shift occurs roughly 70 ka ago and matches the trend we observe in values observed at background points (*k* = 3, *edf* = 2, *p* < 0.001; Supplemental 9).

### Spatiotemporal species distribution model

The spatiotemporal species distribution model incorporates both the spatial and temporal variability present in the archaeological record to estimate the fundamental and potential niche space. Past iterations of species distributions models generally incorporate spatial variation, but location is not limited to space, as time is of central importance in defining a location. Due to the time depth and limited resolution of the paleoclimate data, we are limited in temporal estimation to 1000-years intervals, however this a significant improvement over models that do not incorporate temporal variability or focus on a narrow band of time. It is important to note that this is a single spatiotemporal model. The idea behind the model is that Neanderthals were hunter-gatherers for the entirety of their existence and that between 145 ka and 30 ka ago, there were no significant technological innovations allowing for the expansion of their fundamental climate niche space^45^. Thus, the same underlying processes of a foraging subsistence pattern governed neanderthal land use from 145 ka to 30 ka ago. These assumptions do not mean that there is not variation in archaeological material, both lithic and zooarchaeological, but rather that the same underlying decision structure, limited by the impact tools had on hunting decisions, is present throughout the time period^63^. By constructing a single model where the spatial and temporal variability are represented in the extracted environmental variables, we leverage the small sample size (*n* = 94) to estimate the fundamental and potential^34^ Neanderthal climatic niche spaces. It is important to note that neither time nor spatial location are variables in this model, only the paleoclimate variables observed at archaeological contexts and background points.

To create our spatiotemporal species distribution model, we use a machine learning method common in ecology known as Maximum Entropy (Maxent). Maxent has proven effective for modeling species distributions in both the past, present, and future^36,44,63,93^. Additionally, Maxent’s use of background points to sample the range of environmental values present in a study^88,94^ area and rather conservative predictions make it adept for archaeological applications^36^. We use the package *ENMeval* to construct our spatiotemporal species distribution model and the *maxnet* algorithm^95^. To begin, we test model performance across potential model parameter settings using a ten-fold cross validation. We vary the regularization multiplier (1–5) and the types of feature classes (linear, linear quadratic, linear quadratic hinge, and hinge) allowed (see Supplemental 9). The final model is selected by isolating models that produce *∆* AIC_c_ values less than 2, selecting the models with lowest test omission rate, and breaking any ties with the highest average AUC from the ten-fold cross validation^96–98^. The model selection process identifies a model constructed with a regularization multiplier of 1 and a linear quadratic feature class. Using this model, we create a final model using all the observations (see Supplemental 9).

Using the final model, we are able to the estimate fundamental niche space of Neanderthals (Fig. 3). Characteristics of the fundamental niche estimated in the Results are based on where values for bio01 and bio12 exceed a predicted probability of 0.5.

### Spatiotemporal predictions

Using the final model, we can then predict over the spatiotemporal stack of predictor variables to visualize and measure how the geographically projected potential Neanderthal climatic niche space changed through time. We predict over a spatiotemporal raster stack of Western Eurasia from 350 ka ago to present-day (see Supplemental 9). We produce two sets of predictions. The first is a continuous logistic prediction wherein the values range from 0 to 1 and can be interpreted as the probability that the cell was used by Neanderthals during that time step^50^ or, in our case, as the climatic suitability for Neanderthals. The second is a binary prediction, wherein we use the model evaluation of our final model to identify the continuous prediction value at which we maximize the true positive rate and false negative rate of our predictions. This is the optimal threshold^50^. We reclassify the continuous predictions based on this threshold, with values above being classified as presence and those below as absence. Here, we interpret the binary predictions of presence as representing the projected potential climatic niche space of Neanderthals. Note that this is the projected potential niche space of Neanderthals, thus these are areas where the climatic conditions are deemed suitable for Neanderthals. Whether or not these areas were actually occupied by Neanderthals remains unknown.

The prediction rasters are in the Lambert azimuth equal-area projection (EPSG:3035) with a cell size of 33.5 km × 33.5 km resulting in cells that are 1122.25 km^2^.

### Neanderthal niche space

To estimate the size of the projected potential Neanderthal niche space through time, we sum the number of cells in each millennium predicted as presence. Doing so acts as a measure for the size of the projected potential Neanderthal niche space allowing us to track changes in the projected potential niche space size through time. Using the optimal threshold to estimate the size of the project potential niche space could be over- or underestimating the actual projected potential niche space size of Neanderthals, but it is logical to assume that actual projected potential niche space size scales linearly with our estimates of projected potential niche space size. Using a generalized additive mixed effect model (GAMM) with a Poisson distribution, which uses a generalized additive model, an extension of generalized linear models, to incorporate non-linear responses, and a linear mixed effect model to account for the temporal autocorrelation structure in the data^51^, we test for change in Neanderthal niche space size between 145 ka and 30 ka ago (see Supplemental 9).

## Data Availability

All data and code are available as supplementary information on Zenodo^85^. SI 1—ROCEEH Archaeological Observations. SI 2—ROCEEH Dates Data 1. SI 3—ROCEEH Dates Data 2. SI 4—ROCEEH Dates Data 3. SI 5—Spatiotemporal Archaeological Observations. SI 6—Spatiotemporal Background Points. SI 7—High-Resolution Spatiotemporal Predictions. SI 8—Neanderthal Niche Space 145–30 ka ago. SI 9—Analysis Markdown Document. SI 10—δ18O Record. SI 11—Neanderthal Niche Space 350 ka to Present.
